# Cross-Sectional Surveys of Measles Antibodies in the Jiangsu Province of China from 2008 to 2010: The Effect of High Coverage with Two Doses of Measles Vaccine among Children

**DOI:** 10.1371/journal.pone.0066771

**Published:** 2013-06-25

**Authors:** Yuanbao Liu, Peishan Lu, Ying Hu, Zhiguo Wang, Xiuying Deng, Fubao Ma, Hong Tao, Chengmei Jia, Xiaoyan Ding, Haitao Yang, Pei Liu, Jie Min

**Affiliations:** 1 Department of Expanded Programme on Immunization, Jiangsu Provincial Center for Disease Control and Prevention, Nanjing, Jiangsu Province, China; 2 Department of Biostatistics and Epidemiology, School of Public Health, Southeast University, Nanjing, Jiangsu Province, China; The University of Adelaide, Australia

## Abstract

**Background:**

Changes in the epidemiological characteristics of measles since 2007 appeared in the Jiangsu province. Although the reported coverage with two doses of measles vaccine was greater than 95% in most regions of the province, measles incidence remained high across the whole province. Cross-sectional serological surveys of measles antibodies in the Jiangsu province of China were conducted from 2008 to 2010 to assess and track population immunity.

**Methods:**

Measles-specific IgG levels were measured in serum samples using ELISA. GMTs and seroprevalence with 95% CIs were calculated by region, gender, and age. ANOVA and χ^2^ tests were used to test for statistically significant differences between groups for GMT levels and seroprevalence, respectively.

**Results:**

Seroprevalence showed a significantly increasing trend annually (CMH χ^2^ = 40.32, p<0.0001). Although the seroprevalence among children aged 2–15 years was consistently over 95%, vaccine-induced measles antibodies may wane over time. Measles seropositivity in the Jiangsu province was 91.7% (95% CI: 90.1–93.2%) in 2010. Among adults aged 15 to 29-year-olds, the seropositivity rate was 88.4% (95% CI: 82.7–92.8%).

**Conclusions:**

Vaccination strategies may need to be adjusted depending on the individual age and regions, particularly individuals between the ages of 8 months-14 years old and 20–29 years old. Additional SIAs are likely required to eliminate measles in China.

## Introduction

Measles is a highly contagious, vaccine-preventable disease. A routine two-dose, single-antigen, live attenuated measles vaccine has been available for children administered the first dose in 8–12 months and the second dose at 7 years old in the Jiangsu province of China since 1978. In 1997, the age for the second dose was lowered to 4 years of age [1]. The routine measles vaccination schedule was changed in 2006 to administration of the measles vaccine at 8 months of age followed by the measles, mumps, and rubella (MMR) vaccine at 18–24 months of age. [2]. A Chinese national plan for the elimination of measles was also approved in 2006 that was consistent with the World Health Organization (WHO) initiative to eliminate measles in the Western Pacific Region by the year 2012 [3]. This plan included (1) reaching at least 95% immunity to measles in each cohort born after the adoption of the elimination goal, (2) conducting targeted supplementary immunization activities (SIAs), and (3) strengthening the routine surveillance system for measles. Serological surveillance has been a core component of integrated measles surveillance [4].

Since 2007, changes in the epidemiological characteristics of measles appeared in the Jiangsu province, which may be associated with the increasing size of the migrant population [5]. The highest incidence of measles occurred in children less than 5 years old, especially among children less than 8 months of age. During the same time period, the incidence of measles among adults also increased, with most cases occurring among individuals 20–30 years of age. Catch-up supplemental immunization activities (SIAs) among children from 8 months to 15 years old were conducted in 2009 to achieve high levels of population immunity and rapidly interrupt the chain of measles virus transmission in the province. Follow-up SIAs among children from 8 months to 5 years of age were conducted in 2010.

Measles seroprevalence surveys were conducted in the Jiangsu province from 2008 to 2010 to track changes in population immunity year by year and to identify the susceptible or high-risk cohorts to help target immunization activities. In this study, we report the results and interpretation of those surveys.

## Materials and Methods

### Serological survey

Population-based, cross-sectional surveys for IgG antibodies to measles virus were conducted annually in the Jiangsu province between 2008 and 2010. The 13 cities within the Jiangsu province were stratified into 3 regions (south, center, and north) to account for variations in geography and socioeconomic status. One city in each region was sampled at random. Individuals within each selected city were sampled to be proportionally representative by age and gender.

A total of 10,902 serum samples from individuals 2 months to 74 years old were collected over the 3 study years. Sera were stratified into 10 age groups in each region: ≤7 months, 8–12 months, 13–24 months, 25 months-4 years, 5–9 years, 10–14 years, 15–19 years, 20–29 years, 30–39 years, and ≥40 years old.

Approval for the study was obtained each year from the Medical Ethics Committee of the Jiangsu Provincial Center for Disease Control and Prevention. Written informed consent was signed by individuals or by parents of children. Participants were asked to anonymously fill out a questionnaire about personal information such as sex, age or date of birth, vaccination status, and date of sampling.

### Laboratory assay

Serum samples were stored at −70°C before being tested. Serological tests were performed at the measles laboratory of the Department of Expanded Program on Immunization, Jiangsu Provincial Center for Disease Control and Prevention. This laboratory meets the accreditation criteria for WHO National Measles Laboratories.

Commercial ELISA kits (SERION ELISA classic anti-measles virus IgG, InstitutVirion\Serion GmbH) were used to detect and quantify human IgG antibodies against measles virus in sera. The control and standard sera were ready-to-use without further dilution. For each test run, control and standard sera were included independent of the number of microtiter test strips used, and the standard sera were set up in duplicate. In addition, in-house control samples were included with every assay run. To maintain high quality control, we took strict precautions to avoid non-specific binding throughout the process: avoiding contamination and severe hemolysis during sera collection and separation; prohibited repeated freezing-thawing of serum; leaving the reagent at room temperature for 20 minutes before the start of the test procedure; using aseptic techniques when removing aliquots from reagent tubes; and adequate washing and avoiding foaming.

ELISA results were expressed quantitatively as optical density (OD) measured at 405 nm. Antibody activities (titers, IU/ml) were calculated using software from SERION, and antibody and titers were categorized as negative, equivocal, or positive using fixed cut-off values that were in agreement with international standards [6], [7]. Greater than 0.20 IU/ml was considered positive, less than 0.20 IU/ml was considered negative, and samples with titers between 0.15 IU/ml and 0.20 IU/ml were retested prior to categorization as positive or negative.

### Statistical analysis

Data from the questionnaires were double entered into Epidata software with suitable edit checks and validations. The geometric means titers (GMTs) and seroprevalence of antibodies were calculated with their 95% confidence intervals (95% CIs), stratifying values by region, gender, and age grouping.

Analysis of variance (ANOVA) was used for comparisons of GMTs with a p-value of 0.05 set as the significance threshold. The Student-Newman-Keuls q test (SNK-q test) was used for multiple comparisons. Differences among seroprevalence by region, sex, and age in each year were assessed by Pearson's χ^2^ test with a significance level of 0.05. A Cochran-Mantel-Haenszel χ^2^ test (CMH χ^2^) was used to analyze the trend in seroprevalence between years. R 2.10.0 statistical software was used for analyses [8].

## Results

The GMT of measles antibodies in 2009 was 1.52 IU/ml, which is significantly higher when compared to 2008 (0.80 IU/ml, p<0.05) but not significantly different when compared to 2010 (1.25 IU/ml, p>0.05). The GMT of measles antibodies in 2010 was significantly higher when compared to 2008 (p<0.05, Table 1).

**Table 1:** GMT of measles antibodies by region, sex, and age groups in the Jiangsu province, 2008–2010(IU/ml).

Characteristics		2008			2009			2010		
		NO.	GM	95%CI	NO.	GMT	95%CI	NO.	GMT	95%CI
Region	North	1162	0.73	(0.67,0.79)	1851	1.90**^a^**	(1.79,2.02)	444	0.97**^b,c^**	(0.87,1.08)
	Central	1332	0.64	(0.60,0.69)	1224	1.07**^a^**	(0.98,1.17)	407	1.25**^b,c^**	(1.11,1.42)
	South	2116	0.96	(0.90,1.01)	1934	1.52**^a^**	(1.44,1.60)	432	1.61**^c^**	(1.39,1.87)
Sex	Male	2259	0.75	(0.71,0.80)	2687	1.50**^a^**	(1.42,1.58)	674	1.23**^c^**	(1.11,1.36)
	Female	2351	0.84	(0.80,0.89)	2322	1.54**^a^**	(1.46,1.62)	609	1.28**^c^**	(1.14,1.43)
Age	≤7months	367	0.06	(0.05,0.07)	314	0.09**^a^**	(0.06,0.17)	44	0.13	(0.07,0.26)
	8 m–12 m	183	0.40	(0.30,0.54)	431	0.95**^a^**	(0.80,1.14)	99	1.00**^c^**	(0.69,1.44)
	13 m–24 m	216	1.71	(1.48,1.97)	424	2.54**^a^**	(2.25,2.86)	101	3.00**^c^**	(2.36,3.80)
	25 m–4 y	491	1.86	(1.73,2.01)	905	2.81**^a^**	(2.63,3.01)	166	2.32	(1.93,2.79)
	5 y–9 y	712	1.16	(1.09,1.24)	1011	1.74**^a^**	(1.64,1.84)	295	1.37**^c^**	(1.22,1.53)
	10 y–14 y	376	0.91	(0.82,0.99)	351	1.77**^a^**	(1.61,1.95)	171	1.08**^c^**	(0.94,1.23)
	15 y–19 y	440	0.73	(0.66,0.81)	642	1.27**^a^**	(1.17,1.38)	164	0.83	(0.71,0.97)
	20 y–29 y	565	0.78	(0.71,0.86)	398	1.21**^a^**	(1.07,1.35)	173	1.16**^c^**	(0.92,1.46)
	30 y–39 y	592	0.92	(0.84,1.00)	204	1.23**^a^**	(1.06,1.42)	32	1.28	(0.95,1.73)
	≥40years	668	0.99	(0.92,1.06)	329	1.51**^a^**	(1.37,1.67)	38	1.61**^c^**	(1.19,2.18)
	≥13months	4060	1.04	(1.01,1.07)	4264	1.79 **^a^**	(1.74,1.85)	1140	1.39**^c^**	(1.30,1.49)
Total		4610	0.80	(0.76,0.83)	5009	1.52**^a^**	(1.46,1.57)	1283	1.25**^c^**	(1.16,1.35)

Abbreviations: CI, confidence interval; GMT, geometric mean titer; ^a^ p<0.05 2009 vs. 2008, ^b^ p<0.05 2010 vs. 2009, ^c^ p<0.05 2010 vs. 2008 using ANOVA test, The Student-Newman-Keulsq test was applied for multiple comparison.

Seroprevalence in the entire province showed a significantly increasing trend by year (CMH χ^2^ = 40.32, p<0.0001). While the prevalence of measles antibodies in 2008 (88.7%) was lower than in the later 2 years, there was no statistically significant difference in the prevalence of measles antibodies between 2009 and 2010 (93.6% vs. 91.7%, p>0.05, Table 2).

**Table 2:** Seroprevalence of measles antibodies by region, sex, and age group in the Jiangsu province, 2008–2010 (%).

Characteristics		2008^a^			2009			2010		
		NO.	Seroprevalence^b^	95% CI	NO.	Seroprevalence	95% CI	NO.	Seroprevalence	95% CI
Area	North	1162	86.8	(84.8,88.7)	1851	95.2	(94.1,96.1)	444	91.7	(88.7,94.1)
	Central	1332	87.5	(85.6,89.2)	1224	87.9	(85.9,89.7)	407	90.7	(87.4,93.3)
	South	2116	90.5	(89.2,91.7)	1934	95.6	(94.5,96.4)	432	92.8	(89.9,95.1)
Sex	Male	2259	87.4	(86.0,88.8)	2687	93.2	(92.2,94.1)	674	92.1	(89.8,94.1)
	Female	2351	89.9	(88.6,91.1)	2322	93.9	(92.9,94.9)	609	91.3	(88.8,93.4)
Age	≤7months	367	20.4	(16.4,24.9)	314	36.7	(31.0,42.2)	44	31.8	(18.6,47.6)
	8 m–12 m	183	70.5	(63.3,76.9)	431	82.8	(78.9,86.3)	99	77.8	(68.3,85.5)
	13 m–24 m	216	96.8	(93.4,98.7)	424	96.5	(94.2,98.0)	101	97.0	(91.6,99.4)
	25 m–4 y	491	99.6	(98.5,99.9)	905	98.6	(97.6,99.2)	166	97.6	(93.9,99.3)
	5 y–9 y	712	97.9	(96.6,98.8)	1011	98.6	(97.7,99.2)	295	97.9	(95.6,99.3)
	10 y–14 y	376	95.7	(93.2,97.6)	351	98.1	(95.9,99.2)	171	96.5	(92.5,98.7)
	15 y–19 y	440	92.1	(89.1,94.4)	642	95.9	(94.1,97.3)	164	91.5	(86.1,95.5)
	20 y–29 y	565	92.7	(90.3,94.7)	398	94.2	(91.5,96.3)	173	88.4	(82.7,92.8)
	30 y–39 y	592	93.8	(91.5,95.6)	204	96.6	(93.6,98.6)	32	100.0	-
	≥40years	668	96.7	(95.1,97.9)	329	97.6	(95.3,98.9)	38	97.4	(86.2,99.9)
	≥13months	4060	95.7	(95.0,96.3)	4264	97.4	(96.8,97.8)	1140	95.3	(93.9,96.4)
Total		4610	88.7	(87.8–89.6)	5009	93.6	(92.8–94.2)	1283	91.7	(90.1–93.2)

Abbreviations: CI, confidence interval. ^a^ CMH χ^2^ test was used to test the trend in seroprevalence over years. The seroprevalence of the following stratifications showed a significantly increasing trend over years: north region, south region, male, female, ≤7 months old, and 8–12 months old. ^b^ Pearson's χ^2^ test was used to detect differences between seroprevalence by region, sex, and age group in each year.

### GMT and prevalence of measles antibodies in different regions in 2008–2010

As shown in Tables 1 and 2, the lowest GMT of measles antibodies was observed in the central region in 2008–2009, while the lowest GMT in 2010 was seen in the north region. In 2008, the prevalence of measles antibodies in the north and central region were relatively lower when compared to the south region. The lowest seroprevalence occurred in the central region in 2009. In 2010, the seroprevalences of all of 3 regions were approximately 90%, with no significant differences.

The GMTs of all 3 regions showed an increasing trend year by year. Similarly, the seroprevalence of measles antibodies in the north and south regions also showed an increasing trend by year. In contrast, there was no significant increase in seroprevalence in the central region from 2008 to 2010.

### GMT and prevalence of measles antibodies in different age groups in 2008–2010

The GMTs from sera from children less than 7 months of age were below 0.13 IU/ml in each surveyed year. After the first dose of measles-containing vaccine was administered at 8 months of age, the GMT increased to 1.00 IU/ml; the highest GMT appeared among children from 13 months-4 years old. Most of these children received two doses of measles-containing vaccines. Subsequently, the GMT declined to 1–1.5 IU/ml and was similar in the next age group.

Age-specific seroprevalence showed a trend similar to the GMT in each year: less than 36.7% of children aged ≤7 months were seropositive. Seroprevalence increased to more than 70.5% in children 8–12 months old, and increased further to more than 95% in the 13 months to 4 years old age group. Seroprevalence remained relatively stable through 15 years of age. Seropositivity declined to 88.4% in the age group from 15 to 29 years old, but in adults aged 30 years and older, seropositivity increase to more than 95% and remained high.

Although there were no statistically significant changes in seroprevalence from 2008 to 2010 in any age group older than 8 months, the GMT had an increasing trend in the following age groups: 8–12 months, 13–24 months, 5–9 years, 10–14 years, 20–29 years, and ≥40 years old.

### GMT and prevalence of measles antibodies between sexes from 2008–2010

No significant difference was observed between males and females in either the GMT or the prevalence of protective measles antibodies in the provincial sample each year.

### Region-specific GMT and prevalence of measles antibodies in different age groups in 2008–2010

**Figure 1:**
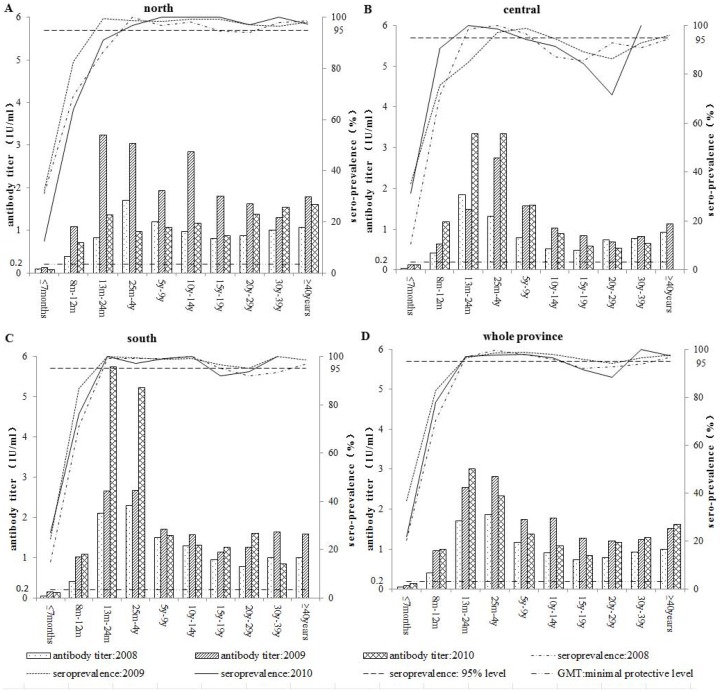
Region-specific geometric mean titers (GMTs) and prevalence of measles antibodies in different age groups from 2008–2010. The seroprevalence in each region showed an upward trend with age: the GMT climbed to a peak between 13 months - 4 years of age followed by a downward trend in subsequent ages (A, B, and C), similar to what was seen throughout the whole province (D). The seroprevalence in the central region declined dramatically in individuals 15–29 years old in 2010 (B).

As Figure 1 shows, although the seroprevalence in each region showed an upward trend with age, GMT peaked between 13 months and 4 years followed by a downward trend (Figure 1A, B, and C), which was similar to that seen in the whole province (Figure 1D). It should be noted that the seroprevalence in central region declined dramatically in individuals aged the 20–29 years in 2010 (Figure 1B).

## Discussion

According to the Western Pacific Regional Plan of Action for Measles Elimination (WHO, January 2003), the elimination of measles can only be achieved if population susceptibility is below the level that can sustain transmission of measles. Thus, 95% population immunity is necessary to interrupt transmission and eliminate measles [3]. Our 3 years of successive cross-sectional surveys revealed that the overall measles seroprevalence was 91.7% in 2010 in the Jiangsu province; this needs to be further increased to achieve the elimination goal. When considering the immunity of the population older than 12 months in age, the seropositive rates for the 3 surveyed years were all over 95%, but there were still wild measles viruses circulating among individuals of these ages. These data suggest that an overall seropositive rate in the province higher than 95% may be insufficient for elimination of measles. For example, the seropositive rate of those older than 12 months was 95.3% in 2010; however, the rate was 88.4% in adults aged 20–29 years old, which may be representative of an epidemic and outbreak of measles due to the frequent migration of adults. Therefore, one of the operational indicators for measles elimination is to maintain 95% immunity to measles in each cohort in every district [3]. Strategies may vary according to population immunity in each age group in each city or county.

The Pan American Health Organization has interrupted indigenous measles transmission since November 2002. One of the primary strategies used to eliminate measles was the strengthening of routine vaccination [9]–[11]. In contrast, the experiences of some European countries showed that SIAs were essential to measles elimination [12]. Our survey supports SIA-based strategy. For instance, although the reported coverage with routine two-dose measles vaccine was over 95% in most regions of the province in 2008, the seroprevalence in several age groups from our survey was lower than 95%, indicating a high risk of measles epidemics to the general population in the province [5]. Further evidence supporting this hypothesis was the significantly higher incidence of measles in the last 2 months of 2008 when compared to 2007.

In February 2009, catch-up SIAs were performed across the whole province among children between 8 months to 15 years old. A total of 9,679,489 children were vaccinated within 2 months, and vaccination coverage of 97.2% was achieved. To accelerate measles elimination and based on the risk assessment of measles epidemics and immunity in the general population, follow-up SIAs in children aged 8 months - 4 years were conducted in October 2010. In that SIA, 4,254,194 children were vaccinated with 95.3% coverage. The most important impacts of SIAs were the upward trend in the immunity of the general population shown in this study, where the measles incidence was observed to dramatically decline from 73.7 per million in 2008 to 9.5 per million in 2010 following the SIA. Several studies also indicated that the estimated reduction in the population susceptible to measles was more than 75% after SIAs [13]–[16].

The purpose of a follow-up campaign is to reduce any accumulation of susceptible individuals born since the previous SIA. These are conducted every 2 - 4 years following catch-up SIAs [17]. The interval between campaigns was only 20 months in Jiangsu province because (1) both rapid, convenient monitoring and coverage surveys after the campaign indicated that the coverage rates in 3 of 13 cities were still lower than 95% in the catch-up SIAs, and (2) the seroprevalence of children from 8 months -15 years old in the central region was lower in 2009 when compared to 2008. The results of the serological survey after the 2010 follow-up campaign revealed that the seroprevalence of 8 month - 4 year olds was significantly higher when compared to 2009 in the central region. In contrast, a small rise in GMT and relatively stable seroprevalence were seen after a follow-up campaign in the other regions.

When the results of these surveys were interpreted longitudinally, seroprevalence and GMTs in children aged 8–12 months (when the first vaccination was offered) was lower than 83% and 1.00 IU/ml, respectively. In children 25 months - 4 years old (when the second vaccination was offered), a significant rise in the prevalence of protective levels of measles antibodies and GMT was observed. These data demonstrated that a two-dose schedule can provide a second chance at immunization for primary vaccine failures and for persons who missed the first dose. The results from other studies showed that short-lived antibodies titers increased after booster immunization with live attenuated vaccine [18], [19], which may represent the immune response when individuals with pre-existing immunity are vaccinated.

Another challenge to the elimination of measles is that, despite the very high seroprevalence among children aged 24 months to 14 years old, the GMTs showed a downward trend with age (Figure 1A, B, C, and D). This could imply that vaccine-induced measles antibodies wane in time, as indicated by previous studies [20]–[23]. Both GMTs and seroprevalence declined in adults from 15–29-year-olds who were born in the post-vaccine era. Gaston De Serres and others recently found indications that there may be a higher risk of measles among high school students when the first dose of measles vaccine is given before 15 months of age [24].

Our study suggests that administering the first dose of measles vaccine at 8 months of age may not be the optimal age at this stage in the elimination of measles. China provides the first dose of measles vaccine at the young age of 8 months. In 2009, the WHO recommended that countries nearing the elimination of measles consider administering the first dose of measles vaccine at 12 months of age. However, prior to changing the age of the first dose of measles vaccine, the WHO recommends that 4 pieces of evidence be considered: (1) the seroconversion rate by age of vaccination, (2) the actual ages of administration for the first dose, (3) projected differences between vaccination rates at 8 and 12 months of age, and (4) the age-specific incidence of measles [25].

Considering all 4 lines of evidence is essential to making an informed policy decision. For example, with more mothers having vaccine-induced antibody levels, the proportions of their children who derive maternally protective levels of antibodies may be lower in the future [26]. Infants will be at a higher risk of being infected before vaccination if measles circulates. In line with our studies, surveillance over the whole province found increasing proportions of infants younger than 8 months old who were infected with measles in recent years.

The incidence of measles in adults aged 20–29 years was also high, at approximately 70–100 per million from 2005–2010 [27]. Some studies have revealed that conducting a revaccination program in secondary school may be a way to improve immunity for adolescents, which may improve the interruption of measles virus circulation in young adults [28], [29]. Most adults aged 30 years and older were born in the pre-vaccine era and thus infected measles. Persistence of protective measles antibodies after natural infection seems to be for life [30].

## Conclusions

Successive serosurveys measured the age-specific immunity profiles of the general population in each region of the Jiangsu province from 2008–2010. These studies revealed that there are some new challenges facing measles elimination. Further actions to increase vaccination coverage in the general population should be implemented to eliminate measles. Routine vaccination of children should also be strengthened, especially in children 8 months -15 years old. Vaccination strategies may be adjusted depending on the ages and regions [26], [31]. It is urgent to explore new, feasible vaccination strategies for adults 20–29 years old, especially in the central region. Targeted SIAs may be essential to close the age-specific immunity gaps. Finally, gathering evidence to support policy decisions on the timing of the first dose should be a priority for China because the country is nearing the elimination of measles.

## References

[1] Chao M, Lixin H, Jing M, Yan Z, Lei C, et al (2011) measles epidemiological characteristics and progress of measles elimintion in China, 2010. chinese Journal of vaccine and immunization 17: 4.

[2] wenyuan Z (2001) Theory of Vaccines and Immunization. Shanghai: Shanghai scientific and technological literature publishing house. 370–384 p.

[3] World Health Organization. Field guidelines for measles elimination. Available: http://www.wpro.who.int/publications/docs/FieldGuidelines_for_MeaslesElimination_0F24.pdf. Accessed 10 October 2012.

[4] Fu C, Xu J, Liu W, Zhang W, Wang M, et al (2010) Low measles seropositivity rate among children and young adults: A sero-epidemiological study in southern China in 2008. Vaccine 28: 8219–8223. 20688039 10.1016/j.vaccine.2010.07.071

[5] Liu YB, Tao H, Ma FB, Lu PS, Hu Y, et al (2011) Sero-epidemiology of measles in general population in Jiangsu province of China: Application of mixture models to interpret the results from a cross-sectional study. Vaccine 29: 1000–1004. 21163248 10.1016/j.vaccine.2010.11.081

[6] Christenson B, Bottiger M (1994) measles antibody - comparison of long-term vaccination titers, early vaccination titers and naturally acquired-immunity to and booster effects on the measles-virus. Vaccine 12: 129–133. 8147093 10.1016/0264-410x(94)90049-3

[7] Chen RT, Markowitz LE, Albrecht P, Stewart JA, Mofenson LM, et al (1990) measles antibody - reevaluation of protective titers. Journal of Infectious Diseases 162: 1036–1042. 2230231 10.1093/infdis/162.5.1036

[8] R D, Core Team. (2009) R: A Language and Environment for Statistical Computing. Vienna, Austria: R Foundation for Statistical Computing. Available: http://www.R-project.org.Accessed 9 May 2012.

[9] PAHO (2005) Measles Elimination Field Guide, Second Edition. Available: http://www.paho.org/english/ad/fch/im/fieldguide_measles.pdf.Accessed 11 June 2012.

[10] Irons B, Dobbins JG, the Caribbean Vaccine Managers (2011) The Caribbean Experience in Maintaining High Measles Vaccine Coverage. The Journal of Infectious Disease 204: S284–288. 10.1093/infdis/jir212 21666175

[11] Parker Fiebelkorn A, Redd SB, Gallagher K, Rota PA, Rota J, et al (2010) Measles in the United States during the Postelimination Era. The Journal of Infectious Disease 202: 1520–1528. 10.1086/656914 20929352

[12] Muscat M, Bang H, Wohlfahrt J, Glismann S, Mølbak K (2009) Measles in Europe: an epidemiological assessment. The Lancet 373: 383–389. 10.1016/S0140-6736(08)61849-8 19131097

[13] Kim SS, Han HW, Go U, Chung HW (2004) Sero-epidemiology of measles and mumps in Korea: impact of the catch-up campaign on measles immunity. Vaccine 23: 290–297. 15530670 10.1016/j.vaccine.2004.07.030

[14] Zhuo J, Geng W, Hoekstra EJ, Zhong G, Liang X, et al (2011) Impact of Supplementary Immunization Activities in Measles-Endemic Areas: A Case Study From Guangxi, China. The Journal of Infectious Disease 204: S455–462. 10.1093/infdis/jir063 21666199

[15] Khetsuriani N, Deshevoi S, Goel A, Spika J, Martin R, et al (2011) Supplementary Immunization Activities to Achieve Measles Elimination: Experience of the European Region. The Journal of Infectious Disease 204: S343–352. 10.1093/infdis/jir074 21666183

[16] Ki M, Choi BY, Oh JK (2003) Measles vaccine effectiveness in elementary school student cohort, 1999–2001, South Korea. American Journal of Epidemiology 157: S48–S48.

[17] Sniadack DH, Mendoza-Aldana J, Jee Y, Bayutas B, Lorenzo-Mariano KM (2011) Progress and Challenges for Measles Elimination by 2012 in the Western Pacific Region. The Journal of Infectious Disease 204: S439–446. 10.1093/infdis/jir148 21666197

[18] Huiss S, Damien B, Schneider F, Muller CP (1997) Characteristics of asymptomatic secondary immune responses to measles virus in late convalescent donors. Clinical and Experimental Immunology 109: 416–420. 9328115 10.1046/j.1365-2249.1997.00137.x PMC1904781

[19] Markowitz LE, Albrecht P, Orenstein WA, Lett SM, Pugliese TJ, et al (1992) persistence of measles antibody after revaccination. Journal of Infectious Diseases 166: 205–208. 1607699 10.1093/infdis/166.1.205

[20] Vyse AJ, Gay NJ, Hesketh LM, Pebody R, Morgan-Capner P, et al (2006) Interpreting serological surveys using mixture models: the seroepidemiology of measles, mumps and rubella in England and Wales at the beginning of the 21st century. Epidemiology and Infection 134: 1303–1312. 16650326 10.1017/S0950268806006340 PMC2870519

[21] Jaber SM (2006) A serological survey of measles, mumps and rubella immunity among school aged children in western Saudi Arabia. Saudi Medical Journal 27: 63–69. 16432596

[22] Andrews N, Pebody RG, Berbers G, Blondeau C, Crovari P, et al (2000) The European Sero-Epidemiology Network: standardizing the enzyme immunoassay results for measles, mumps and rubella. Epidemiology and Infection 125: 127–141. 11057968 10.1017/s0950268899004173 PMC2869578

[23] Chen C-J, Lee P-I, Hsieh Y-C, Chen P-Y, Ho Y-H, et al (2012) Waning population immunity to measles in Taiwan. Vaccine 30: 6721–6727. 22634294 10.1016/j.vaccine.2012.05.019

[24] De Serres G, Boulianne N, Defay F, Brousseau N, Benoit M, et al (2012) Higher Risk of Measles When the First Dose of a 2-Dose Schedule of Measles Vaccine Is Given at 12–14 Months Versus 15 Months of Age. Clinical Infectious Diseases 55: 394–402. 22543023 10.1093/cid/cis439

[25] WHO (2009) Measles Vaccine -WHO position paper. Weekly Epidemiological Record 84: 349–360.19714924

[26] Sudfeld CR, Navar AM, Halsey NA (2010) Effectiveness of measles vaccination and vitamin A treatment. Int J Epidemiol 39: i48–55. 20348126 10.1093/ije/dyq021 PMC2845860

[27] Yuanbao L, Hong T, Hongyu H, Peishan L, Ying H, et al (2010) epidemiological charcteristics of adults measles and influence to elimination of measles in jiangsu province,2005–2008. Chinese Journal of vaccine and immunization 16: 307–309.

[28] Sasaki A, Suzuki H, Sakai T, Sato M, Shobugawa Y, et al (2007) Measles outbreaks in high schools closely associated with sporting events in Niigata, Japan. Journal of Infection 55: 179–183. 17560656 10.1016/j.jinf.2007.04.004

[29] He H, Chen E-f, Li Q, Wang Z, Yan R, et al (2013) Waning immunity to measles in young adults and booster effects of revaccination in secondary school students. Vaccine 31: 533–537. 23159458 10.1016/j.vaccine.2012.11.014

[30] Markowitz LE, Preblud SR, Fine PEM, Orenstein WA (1990) duration of live measles vaccine-induced immunity. Pediatric Infectious Disease Journal 9: 101–110. 2179836 10.1097/00006454-199002000-00008

[31] Bechini A, Boccalini S, Tiscione E, Pesavento G, Mannelli F, et al (2012) Progress towards measles and rubella elimination in Tuscany, Italy: the role of population seroepidemiological profile. Eur J Public Health 22: 133–139. 20880991 10.1093/eurpub/ckq134

